# Supplementary material for: Cyberbullying and Associated Factors in Member Countries of the European Union: A Systematic Review and Meta-Analysis of Studies with Representative Population Samples
Source: Int J Environ Res Public Health. 2022 Jun 15;19(12):7364. doi: 10.3390/ijerph19127364 (PMC9223899; doi:10.3390/ijerph19127364)
Supplement: Supplementary file 1 [file ijerph-19-07364-s001.zip › Document_S1_NOS_scale_adaptation_cross-sectional.pdf]

Newcastle-Ottawa Scale adapted for cross-sectional studies

Selection:

1. Representativeness of the sample:
    a. Truly representative of the average in the target population. * (all subjects or random sampling)
    b. Somewhat representative of the average in the target group. * (non-random sampling)
    c. Selected group of users/convenience sample.
    d. No description of the derivation of the included subjects.

2. Sample size:
    a. Justified and satisfactory (including sample size calculation). *
    b. Not justified.
    c. No information provided

3. Non-respondents:
    a. Proportion of target sample recruited attains pre-specified target or basic summary of non-respondent characteristics in sampling frame recorded. *
    b. Unsatisfactory recruitment rate, no summary data on non-respondents.
    c. No information provided

4. Ascertainment of the exposure (risk factor):
    a. Self-reported. **
    b. Reported by parents/relatives. *
    c. Source non-reported.

Comparability: (Maximum 2 stars)

1. Comparability of subjects in different outcome groups on the basis of design or analysis. Confounding factors controlled.
    a. Data/ results adjusted for relevant predictors/risk factors/confounders e.g. age, sex, grade, etc. **
    b. Data/results not adjusted for all relevant confounders/risk factors/information not provided.

Outcome:

1. Assessment of outcome:
    a. Assessment using validated or reliable questionnaires/scales. **
    b. Assessment using non-validated nor reliable questionnaires/scales. *
    c. No description of assesment methods used.

2. Statistical test:
    a. Statistical test used to analyse the data clearly described, appropriate and measures of association presented including confidence intervals and probability level (p value). *
    b. Statistical test not appropriate, not described or incomplete.

Cross-sectional Studies:
Strong quality: 7-10 points
Moderate quality: 4-6 points
Poor quality: 0 to 3 points

Newcastle-Ottawa Scale adapted for cross-sectional studies

This scale has been adapted from the Newcastle-Ottawa Quality Assessment Scale for cohort studies to perform a quality assessment of cross-sectional studies for the systematic review, “Cyberbullying and associated factors in member countries of the European Union: A systematic review and meta-analysis of studies with representative population samples.”.

We have not selected one factor that is the most important for comparability, because the variables are not the same in each study. Thus, the principal factor should be identified for each study.

As most of the risk factors in our study are sociodemographic and behavioral characteristics, no official records are used to its ascertainment. So we have assigned two stars if risk factors are self-reported and one star if risk factors are reported by parents or other relatives.

In our study, all outcomes are self-reported. So we have specifically assigned two stars for studies in which self-reported outcomes, are assessed with validated or reliable questionnaires/scales and one star is given to the studies that assess the outcome with non-validated nor reliable questionnaires/scales.
