# Supplementary material for: Cyberbullying and Associated Factors in Member Countries of the European Union: A Systematic Review and Meta-Analysis of Studies with Representative Population Samples
Source: Int J Environ Res Public Health. 2022 Jun 15;19(12):7364. doi: 10.3390/ijerph19127364 (PMC9223899; doi:10.3390/ijerph19127364)
Supplement: Supplementary file 1 [file ijerph-19-07364-s001.zip › Table_S1_Full_search_record_and_strategy.pdf]

Registro de búsquedas bibliográficas: Cyberbullying en jóvenes/niños

Databases	Platform/ Access	Search date	References retrieved
Scopus		31/11/2021	206
WOS (Core Collection)		31/11/2021	427
PUBMED		31/11/2021	198
Medline	Ovid	31/11/2021	164
ERIC	Proquest	31/11/2021	181
Sociological Abstracts	Proquest	31/11/2021	166
Psyinfo	Proquest	31/11/2021	738
CINHAL	EbscoHost	31/11/2021	224
Embase	Elsevier	31/11/2021	357
Cochrane Library		31/11/2021	15
TOTAL con duplicados			2676
Duplicados			1163
TOTAL			1513

Términología

Cyberbullying:

Cyberbullying OR “Cyber-bullying” OR “Cyber bullying” OR Cyberbull* OR Cyberaggression OR “Cyber aggression*” OR Cybervictimization OR “Cyber victimization” OR “Cyber victim*” OR “Cyber harassment” OR "Cyber bullied" OR “Online Bullying” OR “Virtual Bullying” OR “Electronic bullying” OR “Cyber deviance” OR “Cyber abuse”.

Jóvenes/Niños:

Adolescen* OR Teen* OR Young OR Youth OR Student* OR Kid OR Kids OR Child*

Países europeos

Europe OR Albania OR Andorra OR Armenia OR Austria OR Azerbaijan OR Belarus OR Belgium OR Bosnia OR Herzegovina OR Bulgaria OR Croatia OR Cyprus OR “Czech Republic” OR Denmark OR Estonia OR Finland OR France OR Georgia OR Germany OR Greece OR Hungary OR Iceland OR Ireland OR Italy OR Kazakhstan OR Kosovo OR Latvia OR Liechtenstein OR Lithuania OR Luxembourg OR Macedonia OR Malta OR Moldova OR Monaco OR Montenegro OR Netherlands OR Norway OR Poland OR Portugal OR Romania OR Russia OR “San Marino” OR Serbia OR Slovakia OR Slovenia OR Spain OR Sweden OR Switzerland OR Turkey OR Ukraine OR “United Kingdom” OR “Great Britain” OR “Vatican City”

Estrategia Medline(OVID)

1. Cyberbullying/ or Cyberbullying.mp.

2. (Cyberaggression or (Cyber adj2 aggression*)).mp.

3. (cybervictimization or (cyber adj2 victim*)).mp.

4. ((cyber adj2 harassment) or (cyber adj2 bullied) or (cyber adj2 deviance) or (cyber adj2 abuse)).mp.

5. (Online Bullying or Virtual Bullying or Electronic bullying).mp.

6. or/1-5

7. *Adolescent/ or adolescen*.mp.

8. (Youth or Young).mp.

9. Child/ or Child*.mp. or (Kid or Kids).mp.

10. Student*.mp.

11. or/7-10

12. exp Europe/

13. (Europe or Albania or Andorra or Armenia or Austria or Azerbaijan or Belarus or Belgium or Bosnia or Herzegovina or Bulgaria or Croatia or Cyprus or Czech Republic or Denmark or Estonia or Finland or France or Georgia or Germany or Greece or Hungary or Iceland or Ireland or Italy or Kazakhstan or Kosovo or Latvia or Liechtenstein or Lithuania or Luxembourg or Macedonia or Malta or Moldova or Monaco or Montenegro or Netherlands or Norway or Poland or Portugal or Romania or Russia or San Marino or Serbia or Slovakia or Slovenia or Spain or Sweden or Switzerland or Turkey or Ukraine or United Kingdom or Great Britain or Vatican City).mp.

14. or/12-13

15. 6 and 11 and 14

Estrategia Pubmed

Search: **((("Adolescent"[Mesh] OR "Young Adult"[Mesh]) OR (Adolescen*[Text Word] OR Teen*[Text Word] OR Young[Text Word] OR Youth[Text Word] OR Child*[Text Word] OR Student*[Text Word] OR Kid[Text Word] OR Kids[Text Word])) AND (("Cyberbullying"[Mesh]) OR (Cyberbullying[Text Word] OR "cyber-bullying"[Text Word] OR "cyber bullying"[Text Word] OR cyberaggression[Text Word] OR "cyber aggression*"[Text Word] OR cybervictimization[Text Word] OR "cyber**

victimization"[Text Word] OR "cyber victim*"[Text Word] OR "cyber harassment"[Text Word] OR "cyber bullied"[Text Word] OR "online Bullying"[Text Word] OR "virtual Bullying"[Text Word] OR "electronic bullying"[Text Word] OR "cyber deviance"[Text Word] OR "cyber abuse"[Text Word]))) AND (("Europe"[Mesh]) OR (Europe[Text Word] OR Albania[Text Word] OR Andorra[Text Word] OR Armenia[Text Word] OR Austria[Text Word] OR Azerbaijan[Text Word] OR Belarus[Text Word] OR Belgium[Text Word] OR Bosnia[Text Word] AND Herzegovina[Text Word] OR Bulgaria[Text Word] OR Croatia[Text Word] OR Cyprus[Text Word] OR Czech Republic[Text Word] OR Denmark[Text Word] OR Estonia[Text Word] OR Finland[Text Word] OR France[Text Word] OR Georgia[Text Word] OR Germany[Text Word] OR Greece[Text Word] OR Hungary[Text Word] OR Iceland[Text Word] OR Ireland[Text Word] OR Italy[Text Word] OR Kazakhstan[Text Word] OR Kosovo[Text Word] OR Latvia[Text Word] OR Liechtenstein[Text Word] OR Lithuania[Text Word] OR Luxembourg[Text Word] OR Macedonia[Text Word] OR Malta[Text Word] OR Moldova[Text Word] OR Monaco[Text Word] OR Montenegro[Text Word] OR Netherlands[Text Word] OR Norway[Text Word] OR Poland[Text Word] OR Portugal[Text Word] OR Romania[Text Word] OR Russia[Text Word] OR San Marino[Text Word] OR Serbia[Text Word] OR Slovakia[Text Word] OR Slovenia[Text Word] OR Spain[Text Word] OR Sweden[Text Word] OR Switzerland[Text Word] OR Turkey[Text Word] OR Ukraine[Text Word] OR United Kingdom[Text Word] OR Great Britain[Text Word] OR Vatican City[Text Word]))

WOS

Adolescen* OR Teen* OR Young OR Youth OR Student* OR Kid OR Kids OR Child* (Topic) and Cyberbullying OR “Cyber-bullying” OR “Cyber bullying” OR Cyberaggression OR “Cyber aggression*” OR Cybervictimization OR “Cyber victimization” OR “Cyber victim*” OR “Cyber harassment” OR "Cyber bullied" OR “Online Bullying” OR “Virtual Bullying” OR “Electronic bullying” OR “Cyber deviance” OR “Cyber abuse” (Topic) and Europe OR Albania OR Andorra OR Armenia OR Austria OR Azerbaijan OR Belarus OR Belgium OR Bosnia OR Herzegovina OR Bulgaria OR Croatia OR Cyprus OR “Czech Republic” OR Denmark OR Estonia OR Finland OR France OR Georgia OR Germany OR Greece OR Hungary OR Iceland OR Ireland OR Italy OR Kazakhstan OR Kosovo OR Latvia OR Liechtenstein OR Lithuania OR Luxembourg OR Macedonia OR Malta OR Moldova OR Monaco OR Montenegro OR Netherlands OR Norway OR Poland OR Portugal OR Romania OR Russia OR San Marino OR Serbia OR Slovakia OR Slovenia OR Spain OR Sweden OR Switzerland OR Turkey OR Ukraine OR “United Kingdom” OR “Great Britain” OR “Vatican City” (Topic) and Articles or Review Articles or Early Access (Document Types)

Scopus

(KEY (cyberbullying OR "Cyber-bullying" OR "Cyber bullying" OR cyberaggression OR "Cyber aggression*" OR cybervictimization OR "Cyber victimization" OR "Cyber victim*" OR "Cyber harassment" OR "Cyber bullied" OR "Online Bullying" OR "Virtual Bullying" OR "Electronic bullying" OR "Cyber deviance" OR "Cyber abuse") AND KEY (adolescen* OR teen* OR young

OR youth OR student* OR kid OR kids OR Child*) AND KEY (europe OR albania OR andorra OR armenia OR austria OR azerbaijan OR belarus OR belgium OR bosnia OR herzegovina OR bulgaria OR croatia OR cyprus OR "Czech Republic" OR denmark OR estonia OR finland OR france OR georgia OR germany OR greece OR hungary OR iceland OR ireland OR italy OR kazakhstan OR kosovo OR latvia OR liechtenstein OR lithuania OR luxembourg OR macedonia OR malta OR moldova OR monaco OR montenegro OR netherlands OR norway OR poland OR portugal OR romania OR russia OR "San Marino" OR serbia OR slovakia OR slovenia OR spain OR sweden OR switzerland OR turkey OR ukraine OR "United Kingdom" OR "Great Britain" OR "Vatican City"))

ERIC

(Cyberbullying OR "Cyber-bullying" OR "Cyber bullying" OR Cyberaggression OR "Cyber aggression*" OR Cybervictimization OR "Cyber victimization" OR "Cyber victim*" OR "Cyber harassment" OR "Cyber bullied" OR "Online Bullying" OR "Virtual Bullying" OR "Electronic bullying" OR "Cyber deviance" OR "Cyber abuse") AND (Adolescen* OR Teen* OR Young OR Youth OR Student* OR Kid OR Kids OR Child*) AND (Europe OR Albania OR Andorra OR Armenia OR Austria OR Azerbaijan OR Belarus OR Belgium OR Bosnia OR Herzegovina OR Bulgaria OR Croatia OR Cyprus OR "Czech Republic" OR Denmark OR Estonia OR Finland OR France OR Georgia OR Germany OR Greece OR Hungary OR Iceland OR Ireland OR Italy OR Kazakhstan OR Kosovo OR Latvia OR Liechtenstein OR Lithuania OR Luxembourg OR Macedonia OR Malta OR Moldova OR Monaco OR Montenegro OR Netherlands OR Norway OR Poland OR Portugal OR Romania OR Russia OR "San Marino" OR Serbia OR Slovakia OR Slovenia OR Spain OR Sweden OR Switzerland OR Turkey OR Ukraine OR "United Kingdom" OR "Great Britain" OR "Vatican City")

Psyinfo

Idem ERIC

Sociological Abstract

Idem ERIC

CINAHL

(Cyberbullying OR “Cyber-bullying” OR “Cyber bullying” OR Cyberaggression OR “Cyber aggression*” OR Cybervictimization OR “Cyber victimization” OR “Cyber victim*” OR “Cyber harassment” OR "Cyber bullied" OR “Online Bullying” OR “Virtual Bullying” OR “Electronic bullying” OR “Cyber deviance” OR “Cyber abuse”) AND (Adolescen* OR Teen* OR Young OR Youth OR Student* OR Kid OR Kids OR Child*) AND (Europe OR Albania OR Andorra OR Armenia OR Austria OR Azerbaijan OR Belarus OR Belgium OR Bosnia OR Herzegovina OR Bulgaria OR Croatia OR Cyprus OR “Czech Republic” OR Denmark OR Estonia OR Finland OR France OR Georgia OR Germany OR Greece OR Hungary OR Iceland OR Ireland OR Italy OR Kazakhstan OR Kosovo OR Latvia OR Liechtenstein OR Lithuania OR Luxembourg OR Macedonia OR Malta OR Moldova OR Monaco OR Montenegro OR Netherlands OR Norway OR Poland OR Portugal OR Romania OR Russia OR “San Marino” OR Serbia OR Slovakia OR Slovenia OR Spain OR Sweden OR Switzerland OR Turkey OR Ukraine OR “United Kingdom” OR “Great Britain” OR “Vatican City”)

EMBASE

('cyberbullying'/exp OR cyberbullying OR 'cyber-bullying'/exp OR 'cyber-bullying' OR 'cyber bullying'/exp OR 'cyber bullying' OR cyberaggression OR 'cyber aggression*' OR cybervictimization OR 'cyber victimization' OR 'cyber victim*' OR 'cyber harassment'/exp OR 'cyber harassment' OR 'cyber bullied' OR 'online bullying'/exp OR 'online bullying' OR 'virtual bullying' OR 'electronic bullying'/exp OR 'electronic bullying' OR 'cyber deviance' OR 'cyber abuse') AND (adolescen* OR teen* OR young OR 'youth'/exp OR youth OR student* OR kid OR kids OR child*) AND ('europe'/exp OR europe OR 'albania'/exp OR albania OR 'andorra'/exp OR andorra OR 'armenia'/exp OR armenia OR 'austria'/exp OR austria OR 'azerbaijan'/exp OR azerbaijan OR 'belarus'/exp OR belarus OR 'belgium'/exp OR belgium OR bosnia OR herzegovina OR 'bulgaria'/exp OR bulgaria OR 'croatia'/exp OR croatia OR 'cyprus'/exp OR cyprus OR 'czech republic'/exp OR 'czech republic' OR 'denmark'/exp OR denmark OR 'estonia'/exp OR estonia OR 'finland'/exp OR finland OR 'france'/exp OR france OR 'georgia'/exp OR georgia OR 'germany'/exp OR germany OR 'greece'/exp OR greece OR 'hungary'/exp OR hungary OR 'iceland'/exp OR iceland OR 'ireland'/exp OR ireland OR 'italy'/exp OR italy OR 'kazakhstan'/exp OR kazakhstan OR 'kosovo'/exp OR kosovo OR 'latvia'/exp OR latvia OR 'liechtenstein'/exp OR liechtenstein OR 'lithuania'/exp OR lithuania OR 'luxembourg'/exp OR luxembourg OR macedonia OR 'malta'/exp OR malta OR 'moldova'/exp OR moldova OR 'monaco'/exp OR monaco OR 'montenegro'/exp OR montenegro OR 'netherlands'/exp OR netherlands OR 'norway'/exp OR norway OR 'poland'/exp OR poland OR 'portugal'/exp OR portugal OR 'romania'/exp OR romania OR 'russia'/exp OR russia OR 'san marino'/exp OR 'san marino' OR 'serbia'/exp OR serbia OR 'slovakia'/exp OR slovakia OR 'slovenia'/exp OR slovenia OR 'spain'/exp OR spain OR 'sweden'/exp OR sweden OR 'switzerland'/exp OR switzerland OR 'turkey'/exp OR turkey OR 'ukraine'/exp OR ukraine OR 'united kingdom'/exp OR 'united kingdom' OR 'great britain'/exp OR 'great britain' OR 'vatican city'/exp OR 'vatican city') AND #2 AND [embase]/lim NOT ([embase]/lim AND [medline]/lim)

Cochrane

(Cyberbullying OR “Cyber-bullying” OR “Cyber bullying” OR Cyberbull* OR Cyberaggression OR “Cyber aggression*” OR Cybervictimization OR “Cyber victimization” OR “Cyber victim*” OR “Cyber harassment” OR "Cyber bullied" OR “Online Bullying” OR “Virtual Bullying” OR “Electronic bullying” OR “Cyber deviance” OR “Cyber abuse”):ti,ab,kw AND (Adolescen* OR Teen* OR Young OR Youth OR Student* OR Kid OR Kids OR Child*):ti,ab,kw AND (Europe OR Albania OR Andorra OR Armenia OR Austria OR Azerbaijan OR Belarus OR Belgium OR Bosnia OR Herzegovina OR Bulgaria OR Croatia OR Cyprus OR “Czech Republic” OR Denmark OR Estonia OR Finland OR France OR Georgia OR Germany OR Greece OR Hungary OR Iceland OR Ireland OR Italy OR Kazakhstan OR Kosovo OR Latvia OR Liechtenstein OR Lithuania OR Luxembourg OR Macedonia OR Malta OR Moldova OR Monaco OR Montenegro OR Netherlands OR Norway OR Poland OR Portugal OR Romania OR Russia OR “San Marino” OR Serbia OR Slovakia OR Slovenia OR Spain OR Sweden OR Switzerland OR Turkey OR Ukraine OR “United Kingdom” OR “Great Britain” OR “Vatican City”):ti,ab,kw
