# Supplementary material for: Cyberbullying and Associated Factors in Member Countries of the European Union: A Systematic Review and Meta-Analysis of Studies with Representative Population Samples
Source: Int J Environ Res Public Health. 2022 Jun 15;19(12):7364. doi: 10.3390/ijerph19127364 (PMC9223899; doi:10.3390/ijerph19127364)
Supplement: Supplementary file 1 [file ijerph-19-07364-s001.zip › Table_S2_Characteristics_Prevalence_Associated_factors.pdf]

Author / Year / Design	Survey	Cyberbullying prevalence (CB, CV, CP, CV-CP, BS)	Associated factors
Bedrosova et al.<br>2022<br>Cross-sectional survey	EU Kids Online 2018<br>Slovakia, Poland and Czech Rep<br>N = 3 855<br>Age: 11–17<br>F: 52.1%<br><br>Czech Republic (n = 2 227; F: 51.3%),<br><br>Poland (n = 928: F: 54.1%)<br><br>Slovakia (n = 700; F: 52.1%).	**Czech Republic (cz)**<br>CV: 18.6%<br>CP: 10.5%<br>BS: 47.5%<br><br>**Poland (pl)**<br>CV: 31.5%<br>CP: 30.6%<br>BS: 53.1%<br><br>**Slovakia (sl)**<br>CV: 6.4%<br>CP: 3.0 %<br>BS: 13.1 %	**Correlations of the factors of ciberbullying (CV; CP; BS) with other variables**<br>• Age<br>• Gender (0= boys)<br>• Internet use: time they spend online during a weekday (β_{CZ}=0.35)<br>• Potential harmful online content: ($\beta_{CZ=}$0.58; β_{PL}=0.49; β_{SL}=0.72)<br>• Emotional problems<br>• Perceived Discrimination due to individual characteristics: (β_{CZ}=0.23; β_{PL}=0.22; β_{SL}=0.27)<br>• Perceived Discrimination due to group membership characteristics: (β_{CZ}=0.18; β_{SL}=0.27)<br>• Friends support: (β_{CZ}=-0.20)
Garitaonandia et al. 2019<br>Cross-sectional survey<br>% Age	Net Children Go Mobile Project 2015<br>Spain<br>N=500<br>Age: 9 and 16 years	CV: 12%<br>9-10 years of age: 8%<br>11-12 years of age: 13%<br>13-14 years of age: 14%<br>15-16 years of age:15%<br>TOTAL : 12%	
Görzig et al.<br>2017<br>Cross-sectional survey	EU Kids Online 2010<br>18 EU countries*<br>N= 15813<br>Age: 9-16 years<br>F: 49.5%; M: 50.5%<br><br>*Belgium; Bulgaria; Czech Republic; Germany; Denmark; Greece; Spain; Finland; France; Hungary; Italy; Netherlands; Norway; Poland; Portugal; Romania; Sweden; UK	**Victims by country**<br>Romania: 15.4%<br>Denmark: 13.1%<br>Sweden: 13%<br>Norway 10.3%<br>UK: 10.2%<br>France: 9.5%<br>Czech Rep: 9.4%<br>Belgium:8.7%<br>Poland: 7%<br>Bulgaria:6.9%<br>Hungary: 6.5%<br>Finland: 5.35<br>Germany: 5.3%<br>Greece: 5.2%<br>Netherlands: 5.1%<br>Spain: 5%<br>Italy: 2.9%<br>Portugal: 2.8%	• Age (OR=1.20)<br>• Gender (female=0=) (OR=0.63)<br>• SES<br>• Crime<br>• GDP (Gross Domestic Product)<br>• Life expectancy: (OR=0.56)<br>• Population density

Author / Year / Design	Survey	Cyberbullying prevalence (CB, CV, CP, CV-CP, BS)	Associated factors
Hamal et al. 2020<br>Cross-sectional survey<br>% By sex<br>% By age	Nationwide Adolescent Health and Lifestyle Survey<br>AHLS 2015<br>Finland<br>N=6698<br>Age: 12-14 (50%) vs 16-18 years (50%)<br>F: 57.15%%; M: 42.8%	CV:12%<br>CP: 8.1%<br><br>Victims by gender and age<br>Gender<br>Female: 13.4%<br>Male: 10%<br><br>Age:<br>Female<br>12-14: 35.1%<br>16-18: 28.7%<br>Male<br>12-14: 23.8%<br>16-18: 12.4%<br><br>Perpetrators by gender and age<br>Age:<br>Female<br>12-14: 25.8%<br>16-18: 19.9%<br>Male<br>12-14: 32.6%<br>16-18: 21.7%	• Age<br>• Gender (boys=0)<br>• Self-reported health<br>• Health complaints (tension. irritation and headaches)<br>Covariates<br>• Parents´education<br>• Family structure<br><br>CV:<br>• Age 12-14 years (OR=1.51 CI95% (1.23-1.84))<br>• Sex: girls (OR=1.27 CI95% (1.04-1.56)).<br>• Poor health (OR= 2.02 CI95% 1.58-2.61)<br>• To have one health complaint (OR=1.78 CI95% 1.46- 2.27)<br>• To have two (OR=2.89 CI95% 2.36-3.55)<br>• To have three health complaints (OR=5.19 CI95% 4.09-6.59)<br>CP:<br>• Age 12-14 years (OR=1.22 CI95% (1.00-1.48))<br>• Sexo: girls (OR=0.48 CI95% (0.39-0.59)).<br>• Poor health (OR= 1.45 CI95% 1.13-1.87)<br>• To have one health complaint (OR=1.29 CI95% 1.03- 1.62)<br>• To have two (OR=1.98 CI95% 1.57-2.51)<br>• To have three health complaints (OR=2.08 CI95% 1.52-2.83)

Author / Year / Design	Survey	Cyberbullying prevalence (CB, CV, CP, CV-CP, BS)	Associated factors
Lindfors et al 2012<br>Cross-sectional survey<br>% By sex<br>% By age	Nationwide Adolescent Health and Lifestyle Survey (AHLS) 2009 Finland<br>N=5516<br>Age: 12-14 (43,8%) vs 16-18 years (56,2%)<br>F: 66%; M: 44%	CV:11%<br>CP: 9%<br>BS:13%<br>Male N=2288 / Female N=3228<br>CV<br>12: 10% / 12:14%<br>14: 13% / 14: 14%<br>16: 9% / 16: 11%<br>18: 9% / 18: 7%<br>CP<br>12: 8% / 12: 5%<br>14: 13% / 14: 13%<br>16: 12% / 16: 8%<br>18: 10% / 18: 4%<br>CV-CP<br>12: 3% / 12: 2%<br>14: 6% / 14: 5%<br>16: 4% / 16: 3%<br>18: 3% / 18: 2%<br>BS<br>12: 11% / 12: 16%<br>14: 10% / 14: 19%<br>16: 10% / 16: 17%<br>18: 10% / 18: 10%	
Legate et al. 2019<br>Cross-sectional survey	Survey the lives of British youth online 2018 England, Scotland and Wales<br>N=1004<br>Age: 14 (49,5%); 15 (50,5%)<br>F: 45,9%; M:53,8%		**Correlations beetween study variables and cyberbyllying**<br>• Female<br>• White<br>• Autonomy-supportive parenting (β=-0.30)<br>• Controlling parenting (β=-0.09)<br>• Shame & Guilt (β=0.12)<br>• Punihsment (β=-0.16)<br>• Adolescent reactance (β=0.44)<br>• Parental Concern (β=0.46).

Author / Year / Design	Survey	Cyberbullying prevalence (CB, CV, CP, CV-CP, BS)	Associated factors
Vazsonyi et al. 2012<br>Cross-sectional survey	EU Kids Online 2010<br>25 EU** countries<br>N= 25142<br>Age: 9-16 years<br>F: 50%; M: 50%<br><br>**Austria, Belgium, Bulgaria, Cyprus, Czech Republic, Denmark, Estonia, Finland, France, Germany, Greece, Hungary, Ireland, Italy, Lithuania, Netherlands, Norway, Poland, Portugal, Romania, Slovenia, Spain, Sweden, Turkey, UK		• Low self control<br>• Online anonymity<br>• Online/ Offline bullying behaviours<br>• Externalizing behaviours<br>• Countries (developmental contexts)<br><br>**CV: (male vs female)**<br>• Offline victimization (β=0.35 vs. β=0.41)<br>• CP (β=0.27 vs. β= 0.27)<br>• Offline perpetration (β=0.17 vs. β=0.18)<br>• Low self-control (β=0.11 vs. β= 0.16)<br>• Externalizing behaviours (β=0.14 vs. β=0.13)<br>**CP: (male vs female)**<br>• Offline victimization (β=0.14 vs. β=0.15)<br>• CV (β=0.27 vs. β= 0.27)<br>• Offline perpetration (β=0.35 vs. β=0.37)<br>• Low self-control (β=0.16 vs. β=0.17)<br>• Externalizing behaviours (β=0.18 vs. β=0.16)
